# Head and Neck Cancer Among U.S. Active Component Service Members, 2010–2024

**Published:** 2026-05-15

**Authors:** Christa E. Goodwin

**Affiliations:** Tri-Service Center for Oral Health Studies, Uniformed Services University, Joint Base San Antonio, Fort Sam Houston, TX

## Abstract

This study utilized de-identified surveillance data to estimate the incidence of head and neck cancer among active component service members (Army, Navy, Air Force, Marine Corps, Coast Guard) from 2010 through 2024. This report updates the June 2021 *MSMR* analysis of oral and pharyngeal cancers (2007-2019) by expanding the case definition to include all head and neck cancers and extending the surveillance period through 2024. There were 549 cases of head and neck cancer diagnosed in the active component military during the 15-year period of analysis. The Army had the highest 15-year incidence rate (3.3 per 100,000 person-years) compared to the Navy (2.6 per 100,000), Air Force (2.6 per 100,000), Coast Guard (2.0 per 100,000), and Marine Corps (1.3 per 100,000). Service members ages 40 years and older had the highest overall incidence rate (12.3 per 100,000), which was 3.3 times the next highest rate observed among those ages 35-39 years. The 15-year male incidence rate (2.9 per 100,000) was greater than that among females (1.7 per 100,000). The parotid gland was the most common site of diagnosis, comprising 14.8% of cases.

**What are the new findings?**

From 2010 through 2024, 549 cases of head and neck cancer were diagnosed among U.S. active component service members. The branch of service, sex, and age group with the highest incidence rates were the Army, males, and those ages 40 years and older. The most common site was the parotid gland.

**What is the impact on readiness and force health protection?**

This report provides the most current head and neck cancer incidence data for active component service members from 2010 through 2024; it establishes baseline rates for monitoring of future trends and highlights specific high-risk populations (e.g., men, Army personnel, service members ages 40 years and older). Although head and neck cancer is the seventh most prevalent cancer worldwide, its incidence among active component service members is seldom reported. Head and neck cancer is often not diagnosed until it has metastasized. Significant physical limitations (e.g., difficulty chewing, speaking, and swallowing) and psychosocial effects (e.g., anxiety, depression, social isolation), compromising service member readiness, can accompany this type of cancer.

Head and neck cancer (HNC), the seventh most prevalent cancer worldwide, ^1 , 2^ is a collective term for cancers originating in the head and neck region including the lip, oral cavity, nasal cavity, paranasal sinuses, salivary glands, pharynx, and larynx. In 2022, there were over 940,000 new cases of HNC and 480,000 fatalities globally. ^1 , 2^ Signs and symptoms may vary depending upon where specifically within the head and neck region the cancer originated. Cancer originating in the oral cavity may result in sores that do not heal, growth or swelling causing dentures to fit poorly, and unusual bleeding or pain. ^3 , 4^ Alternatively, cancer that originates in the pharynx may cause difficulty with essential functions including breathing, speaking, and swallowing food. ^3 , 4^ Various factors dictate the course of treatment including the location and stage of the tumor, as well as patient-specific factors, such as age and health history. Considering the complexity of this type of cancer and the numerous subsites, a multidisciplinary approach to treatment is required. ^5^

Survival rates, like course of treatment, are strongly influenced by the location and stage at diagnosis. According to the National Cancer Institute's Surveillance, Epidemiology, and End Results Program, the overall 5-year relative survival rate of oral cavity and pharynx cancer is 69.5%. ^6^ When considering the stage at diagnosis, the 5-year survival rates for localized (i.e., confined to primary site), regional (i.e., spread to regional lymph nodes), and distant (i.e., metastasized) oral cavity and pharynx cancer are 88.4%, 69.4%, and 36.9%, respectively. ^6^ Laryngeal cancer survival rates, however, are lower compared to oral cavity and pharynx cancer. ^7^ The overall 5-year relative survival rate is 62.1%, and the 5-year survival rates for localized, regional, and distant laryngeal cancer are 79.3%, 49%, and 35.2%, respectively. ^7^

Major risk factors of HNC include alcohol and tobacco use, betel nut chewing, and infection with human papillomavirus (HPV). ^8^ HPV infections are usually asymptomatic and resolve naturally. However, long-lasting infections with high-risk strains of HPV (16 and 18) can cause various types of cancer (e.g., anal, cervical, oropharyngeal, penile, vaginal, vulvar). ^9 , 10^ Remarkably, about 70% of oropharyngeal squamous cell carcinoma cases in the U.S. are attributed to HPV. ^8 , 10 , 11^

Additional risk factors of HNC include genetics, Epstein-Barr virus infection, radiation therapy, exposure to occupational or environmental carcinogens, and immunodeficiency. ^8^ HNC is more likely to be diagnosed in those older than age 55 years and is more than twice as common in men compared to women. ^3 , 12 , 13^ Moreover, a recent study determined that men are far more susceptible to HNC compared to women, regardless of tobacco and alcohol use. ^13^

Numerous studies have explored the incidence of HNC among military veterans; the same cannot be said about active component service members (ACSMs). This is unsurprising, as the active component military population is considered a generally young population, and HNC is significantly more common among those older than age 55 years. A recent study investigated the incidence of the 16 most common cancers among ACSMs ^14^ ; HNC was not included in that list. This type of cancer represents a small component of the overall cancer burden among this population and, consequently, may not often be explored. However, HNC is usually not diagnosed until it has metastasized. HNC can be accompanied by significant physical limitations (e.g., difficulty chewing, speaking, and swallowing) and psychosocial effects (e.g., anxiety, depression, social isolation), compromising service member readiness. Investigating its incidence among ACSMs is important and relevant. This report serves as an update to the June 2021 *MSMR* analysis on the incidence of oral cavity and pharynx cancers among ACSMs from 2007 through 2019, expanding the case definition to include all HNCs and extending the surveillance period to 2010-2024. ^15^

## Methods

This investigation was completed at the Tri-Service Center for Oral Health Studies (TSCOHS), a center of the Uniformed Services University (USU), the nation's federal health professions academy. The USU Human Research Protection Program approved this study, as protocol DBS.2025.827. Data were obtained from the Armed Forces Health Surveillance Division (AFHSD), the central epidemiological health resource for the U.S. military.

The surveillance population included ACSMs of the U.S. Army, Navy, Air Force, Marine Corps, and Coast Guard diagnosed with HNC from the January 1, 2010 through December 31, 2024. Cases were identified by International Classification of Diseases, 9th and 10th revisions (ICD-9/ICD-10), codes for malignant neoplasms of the lip, oral cavity, pharynx, nasal cavity, larynx, and sinuses **(Table 1)** . This report builds upon a prior 2021 analysis ^15^ of the same population (with the addition of the Coast Guard) from 2007 to 2019 but uses a broader case definition. The prior study was restricted to malignant neoplasms of the lip, oral cavity, and pharynx.

**TABLE 1.** ICD-9 and ICD-10 Diagnostic Codes Used to Identify Head and Neck Cancer Cases, U.S. Active Component Service Members, 2010–2024

ICD-9	ICD-10	Site
140.0 – 149.9	C00.0 – C14.8	Malignant neoplasm of lip, oral cavity, and pharynx
160	C30.0	Malignant neoplasm of the nasal cavity
160.2 – 160.5	C31.0 – C31.3	Malignant neoplasm of the maxillary sinus, ethmoidal sinus, frontal sinus, and sphenoid sinus
161.0 – 161.9	C32.0 – C32.9	Malignant neoplasm of larynx

Abbreviations: ICD-9, International Classification of Diseases, 9th Revision; ICD-10, International Classification of Diseases, 10th Revision.

For surveillance purposes, a case of HNC was defined by AFHSD as either 1 hospitalization with a case defining diagnosis of HNC **(Table 1)** in the first diagnostic position; or 1 hospitalization with a procedure code indicating radiotherapy, chemotherapy, or immunotherapy treatment in the first diagnostic position and a case defining diagnosis of HNC **(Table 1)** in the second diagnostic position; or 3 or more outpatient medical encounters within a 90-day period, with a case-defining HNC diagnosis **(Table 1)** in the first or second diagnostic position. For those who met the case definition, the incidence date was the date of the first qualifying hospitalization or outpatient medical encounter with a case-defining HNC diagnosis. An individual was considered an incident case once per lifetime. Additional variables evaluated included branch of service, sex, year of diagnosis, and age at diagnosis. Annual incidence rates (IRs) were calculated for each service branch, sex, and age group, by dividing the number of cases in that subgroup by the number of ACSMs reported in the Defense Medical Epidemiology Database (DMED) for that subgroup and year. The ACSM population counts reported by DMED are the cumulative person years (p-yrs) contributed during the calendar year of interest for the population substratum.

## Results

**FIGURE 1.**
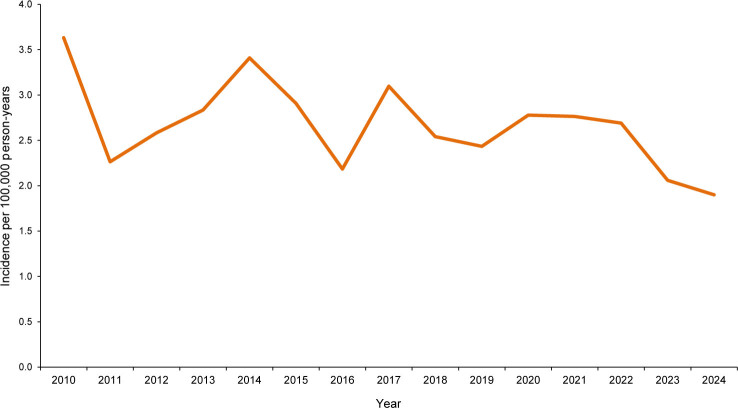
Incidence Rates of Head and Neck Cancer, U.S. Active Component Service Members, 2010–2024

From 2010 through 2024, 549 cases of HNC were diagnosed among U.S. ACSMs. Yearly IRs ranged from 1.9 to 3.6 cases per 100,000 p-yrs **(Figure 1)** with an overall IR of 2.7 cases per 100,000 p-yrs. The number of HNC cases among male service members (n=492) was far greater than that among female service members (n=57) **(Table 2)** . Likewise, the overall IR among men (2.9 per 100,000 p-yrs) exceeded that of women (1.7 per 100,000 p-yrs). As of 2024, male service members accounted for 82.2% of the active component and an even greater proportion (89.6%) of all identified HNC cases during the study period **(Table 2)** .

**TABLE 2.** Counts, Rates, Percentages of All Head and Neck Cancer Cases, U.S. Active Component Service Members, 2010–2024, and Categorical Percentages of the Active Component, 2024

	No.	Rate ^a^	% of Total Cases 2010–2024	% of AC 2024
Total	549	2.7	100	—
Sex				
Male	492	2.9	89.6	82.2
Female	57	1.7	10.4	17.8
Age group, *y*				
<20	3	0.2	0.5	6.6
20–24	61	0.9	11.1	30.0
25–29	74	1.5	13.5	23.4
30–34	61	1.8	11.1	16.7
35–39	89	3.7	16.2	12.9
40+	261	12.3	47.5	10.5
Branch of service				
Army	247	3.3	45.0	34.1
Navy	128	2.6	23.3	25.2
Air Force	127	2.6	23.1	24.8
Marine Corps	35	1.3	6.4	12.9
Coast Guard	12	2.0	2.2	3.1

Abbreviations: No., number; AC, active component; *y* , years.

a Incidence rate per 100,000 person-years.

The largest number (31) of cases occurred among service members who were age 43 years at diagnosis. When evaluated by age group, service members ages 40 years and older accounted for the largest proportion (47.5%) of HNC cases, with the largest overall IR (12.3 per 100,000 p-yrs) compared to the remaining younger age groups **(Table 2)** . As of 2024, service members ages 40 years and older comprised only 10.5% of the active component; the majority (60%) of the active component is younger than age 30 years.

The Army had the largest number of HNC cases (n=247), followed by the Navy (n=128), Air Force (n=127), Marine Corps (n=35), and finally Coast Guard (n=12) **(Table 2)** . The Army accounts for the greatest proportion (34.1% in 2024) of the active component and had an even greater proportion of all cases (45%). Likewise, the Coast Guard constitutes the smallest proportion (3.1% in 2024) of the active component and had the smallest proportion (2.2%) of all cases. The Coast Guard did not have the lowest overall IR, however, which was evidenced by the Marine Corps, with an IR of 1.3 cases per 100,000 p-yrs; the Army had the highest overall IR (3.3 per 100,000 p-yrs) **(Table 2)** .

**FIGURE 2.**
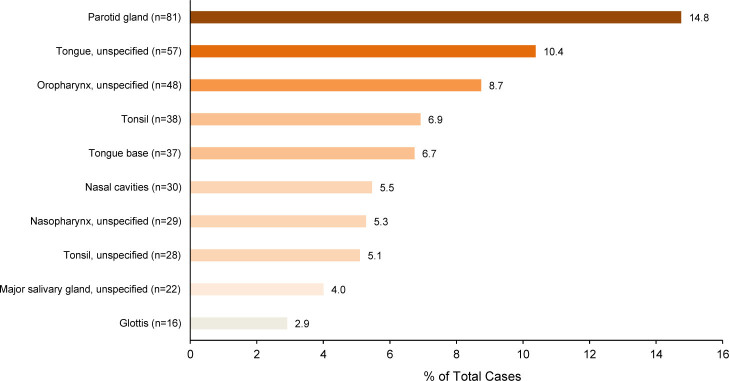
Ten Most Frequent Sites of Head and Neck Cancer, by Percentage of Total Cases, U.S. Active Component Service Members, 2010–2024

The 10 most frequent sites diagnosed with HNC are presented in **Figure 2** . ‘Unspecified’ indicates that the subsite was not documented. For instance, ‘tongue, unspecified’ signifies that the specific location of the tumor on the tongue (e.g., border, dorsal, base) is unknown. The greatest number of cases occurred in the parotid gland (n=81), accounting for 14.8% of all cases during the 15-year surveillance period. However, if combining cases diagnosed in the same primary location, the greatest number of cases (n=94, or 17.1%) occurred on the tongue (tongue, unspecified = 57; tongue base = 37).

## Discussion

This study utilized de-identified surveillance data from AFHSD to estimate the incidence of HNC among ACSMs (Army, Navy, Air Force, Marine Corps, Coast Guard) from 2010 through 2024. This is the first time this group of cancers has been investigated by TSCOHS. These data can help guide military public health policy and future research into specific occupational or lifestyle risk factors within high-risk demographic groups.

In the general population, risk of diagnosis of HNC significantly increases with age; the same can be said for the military. Service members ages 40 years and older comprise the smallest proportion of the active component, yet had the greatest proportion of cases and an IR 3.3 times the next highest IR, which was observed among those ages 35-39 years. Likewise, men in the general population are at a greater risk of developing HNC, which also applies to the military, as male service members had an IR 1.7 times that of female service members.

The 2021 study conducted determined the incidence of oral cavity and pharynx cancer among ACSMs from 2007 through 2019. ^15^ While the present study expanded the 2021 study to include cancer in all locations considered HNC, in addition to the oral cavity and pharynx, very similar results were found. As with the present study, the 2021 investigation determined that the branch of service, sex, and age group with the highest IRs included the Army, men, and those ages 40 years and older. ^15^ Furthermore, the most common diagnosed site in both studies was the parotid gland. ^15^

The difference in IRs among the service branches may be due to the differing age distributions among them, as also suggested in the 2021 study. ^15^ Service members with the greatest risk of HNC were those ages 40 years and older. The Marine Corps had the lowest overall HNC IR. Likewise, the proportion of service members ages 40 years or older is lowest in the Marine Corps (4.9%). Notably, this proportion is at least 2 times lower than that of any other service branch, ranging from 9.9% in the Air Force to 18.3% in the Coast Guard (DMED).

The small number of cases in this study precluded meaningfully stratified analysis due to limited statistical power to separate true trends from random fluctuations. The reliance on de-identified medical encounter data presented with additional limitations. Cancer diagnoses could not be independently verified, and thus, the results could be subject to misclassification errors leading to either an over- or underestimation of cases. Individual risk factors could not be linked to diagnoses; differences in known contributing risk factors (e.g., tobacco and alcohol use, HPV infection) may explain the differences in IRs among the service branches. The Army had the highest IR, 2.5 times that of the Marine Corps, which might suggest higher rates of contributing risk factors in the Army. According to the *2018 Health Related Behavior Survey* , however, both binge and heavy drinking were the highest among Marine Corps and Navy members. ^16^ Furthermore, Marine Corps members were more likely to be current cigarette smokers, electronic cigarette smokers, and smokeless tobacco users compared to service members of all other branches. ^16^

Certain military service members are subject to an added environmental hazard associated with HNC: burn pit exposure. The Department of Veterans Affairs has recognized HNC, in addition to various other cancers, as a “presumptive cancer” related to burn pit exposure among those who served in Iraq, Afghanistan, or certain other areas. ^17^ As such, these individuals may be eligible for disability compensation. ^17^

Risk of developing HNC is related to prolonged, repeated exposure to the known risk factors; this type of cancer can take years, even decades, to develop. ^3 , 4 , 8 - 12^ Considering that HNC diagnosis may not occur until long after a service member has left service, numerous studies have evaluated HNC among the veteran population. Unfortunately, the prevalence of HNC among veterans is nearly twice that of the general population. ^18 - 21^ Factors believed to contribute to this are the high rates of tobacco and alcohol use among this population, as well as low rates of HPV vaccination, ^22 - 26^ which may not have been an option for some veterans, depending upon their ages.

HNC represents a small portion of the overall disease burden among ACSMs compared to other cancers such as breast cancer or melanoma. ^14^ The significance of such a debilitating, albeit uncommon, disease should not be discounted, however. Early detection is paramount to improving prognosis, as is educating patients regarding the signs, symptoms, and risk factors for HNC. ^27 , 28^ Military dentists contribute significantly to early detection. ACSMs are required to have a yearly dental examination which provides dentists with the opportunity to not only educate ACSMs about HNC but evaluate them for suspicious lesions in the head and neck region. This is consistent with the American Dental Association's “Early Detection and Prevention of Oral and Oropharyngeal Cancer” policy, which recommends cancer prevention education and a routine visual and tactile examination for all patients. ^27^ Nevertheless, given the implications of early detection, regular self-examinations for signs of HNC are equally vital.
